# Efficacy and safety of *Lacticaseibacillus rhamnosus* R0011 and *Lactobacillus helveticus* R0052 as an adjuvant for *Helicobacter pylori* eradication: a double-blind, randomized, placebo-controlled study

**DOI:** 10.3389/fgstr.2023.1245993

**Published:** 2023-09-14

**Authors:** Anya Kiattiweerasak, Natsuda Aumpan, Soonthorn Chonprasertsuk, Bubpha Pornthisarn, Sith Siramolpiwat, Patommatat Bhanthumkomol, Pongjarat Nunanan, Navapan Issariyakulkarn, Varocha Mahachai, Yoshio Yamaoka, Ratha-korn Vilaichone

**Affiliations:** ^1^ Gastroenterology and Hepatology Center, Chulabhorn Hospital, Chulabhorn Royal Academy, Bangkok, Thailand; ^2^ Center of Excellence in Digestive Diseases and Gastroenterology Unit, Department of Medicine, Thammasat University, Pathumthani, Thailand; ^3^ Department of Medicine, Chulabhorn International College of Medicine (CICM) at Thammasat University, Pathumthani, Thailand; ^4^ Department of Environmental and Preventive Medicine, Oita University Faculty of Medicine, Yufu, Japan; ^5^ Research Center for Global and Local Infectious Diseases, Oita University, Yufu, Japan; ^6^ Department of Medicine, Gastroenterology and Hepatology Section, Baylor College of Medicine, Houston, TX, United States

**Keywords:** *Helicobacter pylori*, triple therapy, probiotic, gastritis, Thailand

## Abstract

**Background:**

*Helicobacter pylori* eradication is recommended as a way of providing symptomatic relief for dyspepsia. The limited efficacy of triple therapy is a major problem in many countries, including Thailand. Some probiotics have been shown to improve the *H. pylori* eradication rate and reduce side effects. This study aimed at evaluating the efficacy of probiotic (Lacidofil^®^ STRONG) as adjuvant to standard triple therapy.

**Methods:**

This randomized, double-blind, placebo-controlled study was conducted between July 2020 and June 2022. Eligible patients with *H. pylori* gastritis (i.e., *n*=90 out of the 160 patients screened) were randomized to receive 14-day standard triple therapy either with probiotics or with a placebo (N=45/group). The treatment regimen entailed 30 mg lansoprazole administered twice daily, 1,000 mg amoxicillin administered twice daily, and 1 g clarithromycin modified-release formulation administered once daily. A probiotic capsule containing *Lacticaseibacillus rhamnosus* R0011 *and Lactobacillus helveticus* R0052 (Lacidofil^®^ STRONG) or placebo were given twice daily during the eradication therapy and for an additional 4 weeks. Successful *H. pylori* eradication was defined as a negative ^13^C-urea breath test at least 4 weeks after complete eradication.

**Results:**

As per-protocol analysis, eradication rates after the 14-day regimen with probiotic or placebo were 90.9% and 75.0% (*p*=0.047), respectively. Antibiotic susceptibility testing demonstrated high clarithromycin resistance (24%). For clarithromycin-resistant strains, there was no statistical difference in eradication rates between the probiotic and placebo groups. Furthermore, probiotic supplementation significantly reduced treatment side effects, including bloating (OR 0.27 [95% CI 0.10 to 0.75], *p*=0.012), diarrhea (OR 0.23 [95% CI 0.28 to 0.65], *p*=0.006), nausea (OR 0.05 [95% CI 0.01 to 0.36], *p*=0.003), and bitter taste (OR 0.14 [95% CI 0.03 to 0.69], *p*=0.015). In addition, the probiotic group had lower gastrointestinal symptom rating scale (GSRS) scores (1.46 ± 0.36 vs. 2.65 ± 0.66, *p*<0.001) and higher SF-36 health-related quality-of-life scores (63.3 ± 10.2 vs. 57.3 ± 13.4, *p*=0.020) after treatment than the placebo group.

**Conclusion:**

The probiotic adjuvant with 14-day standard triple therapy improved the *H. pylori* eradication rate. Supplementation with Lacidofil^®^ STRONG during the 2-week eradication treatment and 4-week follow-up phase can help to reduce the gastrointestinal side effects of eradication therapy and increase patients’ general health-related quality of life.

## Introduction

*Helicobacter pylori*, a gram-negative group of bacteria, is one of the most common causes of persistent bacterial infection leading to chronic gastritis, peptic ulcer disease, mucosa-associated lymphoid tissue lymphoma, and gastric cancer (1). The early diagnosis and efficient eradication of *H. pylori* infections are crucial for gastric cancer prevention (2), which is particularly relevant in populations where the prevalence of *H. pylori* infection and gastric cancer is high, such as in Asian countries. The prevalence of *H. pylori* infections in Asia was approximately 55% and gastric cancer cases in this region constituted nearly 75% of global cancer deaths (3, 4). Thailand, a country located in the center of Southeast Asia, has recently seen a decline in its *H. pylori* eradication efficacy, with the standard triple therapy recommended as a first-line regimen. According to the Thailand consensus in 2015, standard triple therapy was recommended as a first-line regimen because of its simplicity. However, due to the rise in clarithromycin resistance, the efficacy of the 14-day triple therapy has become largely suboptimal, and it is associated with average eradication rates of approximately 80% or less (5). Several strategies, such as the use of potent acid suppressive medication or supplementation with an adjuvant, were reported to improve the eradication rate of this first-line regimen.

Probiotics are defined as “live microorganisms that, when administered in adequate amounts, confer health benefits on the host” (6). The addition of certain probiotics to *H. pylori* eradication regimens was shown to decrease the side effects of antibiotics (e.g., abdominal pain, nausea, and diarrhea), resulting in improved patient adherence and increased eradication rates (5). Moreover, studies have demonstrated that *Lactobacillus*, the *Bifidobacterium* species, and *Saccharomyces boulardii* can provide beneficial therapeutic effects against *H. pylori* through their modulation of immune responses (7, 8). A recent network meta-analysis reported that *Lactobacilli* and multi-strain formulations yielded superior eradication rates (9). *L. rhamnosus* R0011 and *L. helveticus* R0052 can help to maintain epithelial barrier function, inhibit pathogen adhesion, and downregulate proinflammatory cytokines (10). Compared with different strains of the same species, *L. rhamnosus* R0011 is less likely than *L. rhamnosus* GG to cause bacteremia GG due to the absence of *spaCBA*-encoded pili, which are important adhesins facilitating attachment to the mucosal surface (11). In the present study, we hypothesized that R0011 and R0052 might reduce the severity of *H. pylori* infection and decrease the gastrointestinal side effects typically associated with *H. pylori* eradication regimens.

## Materials and methods

### Patients

Dyspeptic patients aged 18–65 years who underwent upper gastrointestinal (GI) endoscopy at Thammasat University Hospital between July 2020 and June 2022 were assessed for eligibility. Patients diagnosed with *H. pylori* infection were included in this study. The exclusion criteria were the following: previous *H. pylori* treatment, upper GI bleeding, peptic ulcers, gastric cancer, severe comorbidities (e.g., end-stage renal disease, advanced cirrhosis, cancer), contraindication to endoscopic biopsy, current use of probiotic, anticoagulant, or clopidogrel, allergic to penicillin, clarithromycin, or lansoprazole, pregnant women, or unwillingness to participate in the study.

### Diagnosis of *H. pylori* infection

Four biopsies from the antrum and body of stomach were conducted during the upper GI endoscopy and sent for rapid urease test, *H. pylori* culture, and histologic examination. *H. pylori* infection was defined as a positive result to any one of these three diagnostic methods. Endoscopic findings were classified using the updated Sydney system (12).

### Antibiotic susceptibility testing

Antibiotic susceptibility testing was conducted using the Epsilometer test (E-test), or GenoType^®^ HelicoDR. The E-test strip with each antibiotic was placed on the inoculated plate and examined for the subsequent 3–5 days to determine the minimum inhibitory concentrations (MIC) value, defined as the lowest concentration of each antibiotic that can prevent visible bacterial growth. Resistant strains were defined by MIC values of >0.125 mg/L for amoxicillin, > 0.5 mg/L for clarithromycin, >8 mg/L for metronidazole, >1 mg/L for levofloxacin, and > 1 mg/L for tetracycline, as reported by the European Committee on Antimicrobial Susceptibility Testing (13). GenoType^®^ HelicoDR is a form of molecular genetic testing that provides detection of mutation leading to identification of clarithromycin (*rrl* gene) and fluoroquinolone (*gyrA* gene) resistance. The *H. pylori* ATCC 43504 strain was used as a quality control for both antibiotic susceptibility testing and the GenoType^®^ HelicoDR test.

### Therapeutic regimens

**Figure 1:**
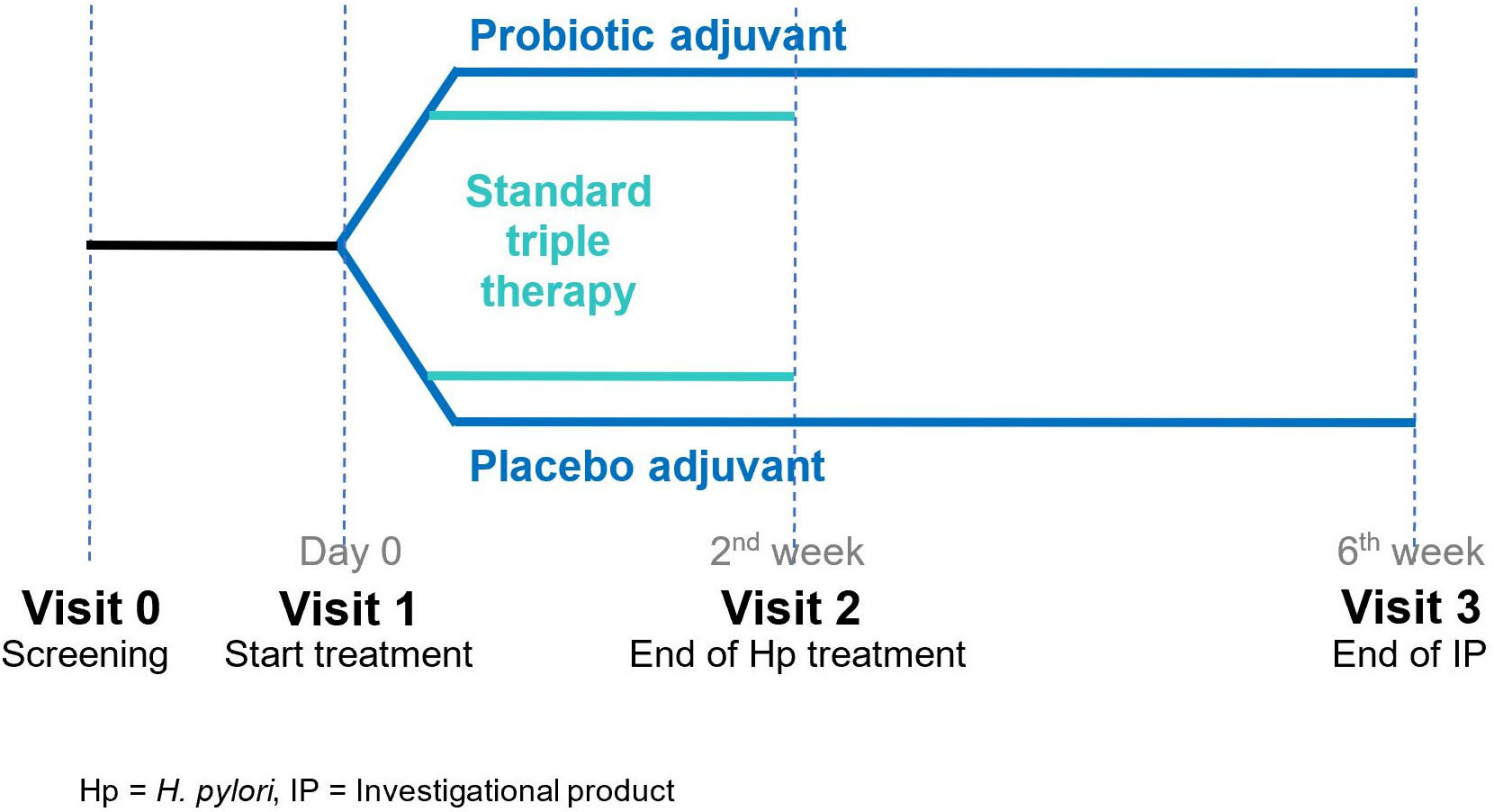
A schematic diagram of therapeutic regimens.

All patients were randomized 1:1 into two groups using a computer-generated list: those in group (1) would undergo 14-day standard triple therapy with probiotic, and those in group (2) would undergo14-day standard triple therapy with placebo. Standard triple therapy consisted of 30 mg lansoprazole taken twice daily before meals, 1,000 mg amoxicillin taken twice daily after meals, and 1 g modified-release formulation of clarithromycin taken once daily after a meal for 14 days. The probiotic capsules (Lacidofil^®^ STRONG) contained four billion colony-forming units of *L. rhamnosus* R0011 and *L. helveticus* R0052, with excipients (maltodextrin, magnesium stearate, and ascorbic acid). Placebo capsules contained only the excipients and were sensorially identical to the probiotic capsules in appearance, smell, and taste. The probiotic adjuvant was administered at a dose of one capsule twice daily after meals during the standard triple eradication treatment and for 4 weeks after, for a total of 42 days (**Figure 1**). Probiotic and placebo capsules were blinded to both the physician and the patients.

### Study outcomes

The primary endpoint in this study was the *H. pylori* eradication rate in the standard triple therapy with probiotics, and standard triple therapy without probiotics, groups. Successful eradication was defined as a negative ^13^C-urea breath test (UBT) at 4 weeks after completion of the 14-day triple therapy (week 6). Participants were not permitted to use proton pump inhibitors in the 2 weeks, or any other antibiotics, in the 4 weeks before undergoing the ^13^C-UBT. The primary endpoint was analyzed using intention-to-treat (ITT) and per-protocol (PP) analyses. The ITT analysis included all randomized participants. Patients who were lost to follow-up were regarded as treatment failures in the ITT analysis. The PP analysis included participants who took at least 80% of study intervention (>11 days of triple therapy with probiotic or placebo) and at least 80% of days within the maintenance period (>22 days of probiotic or placebo in the maintenance period). Patient compliance was assessed by questionnaire and pill count at completion of the triple therapy (week 2) and after the maintenance period (week 6).

The secondary endpoints were to compare the frequency of side effects between the probiotic and placebo groups including gastrointestinal symptoms using the gastrointestinal symptom rating scale (GSRS), and general health-related quality-of-life using the 36-item short form survey (SF-36). GSRS and SF-36 scores were recorded at baseline, after *H. pylori* eradication treatment (week 2), and after completion of the probiotic course (week 6). Adverse events were assessed by personal interview with open-ended questions. Treatment-related adverse events were defined as unexpected new symptoms or the worsening of preexisting symptoms during the treatment period. An adverse event resulting in hospitalization was defined as a serious adverse event. Diarrhea was defined as the passage of unformed stools either ≥ three times or > 250 g per day (14).

### Sample size calculation

Sample size was calculated based on the primary endpoint, assuming an eradication rate of 87% (15) and 65% (16), respectively, for the probiotic and placebo groups. At least 78 patients would be required in the superiority trial to achieve 90% statistical power at a 5% level of significance. Assuming a follow-up loss of 10%, 84 patients (42 patients in each group) were required in this study.

### Statistical analysis

All data were analyzed using SPSS version 22 (SPSS Inc., Chicago, IL, USA). Categorical variables were analyzed using the Fisher’s exact or chi-squared test where appropriate. Continuous variables were analyzed using Student’s *t*-test and reported as the mean ± standard deviation (SD). All *p*-values were two-sided, with *p* < 0.05 considered as the statistical significance threshold. This study was approved by the Human Research Ethics Committee of Thammasat University, Thailand (ClinicalTrials.gov identifier: NCT04473079), and was conducted in accordance with the ICH-GCP guidelines and the Declaration of Helsinki. Informed consent was obtained from all patients in this study.

## Results

**Figure 2:**
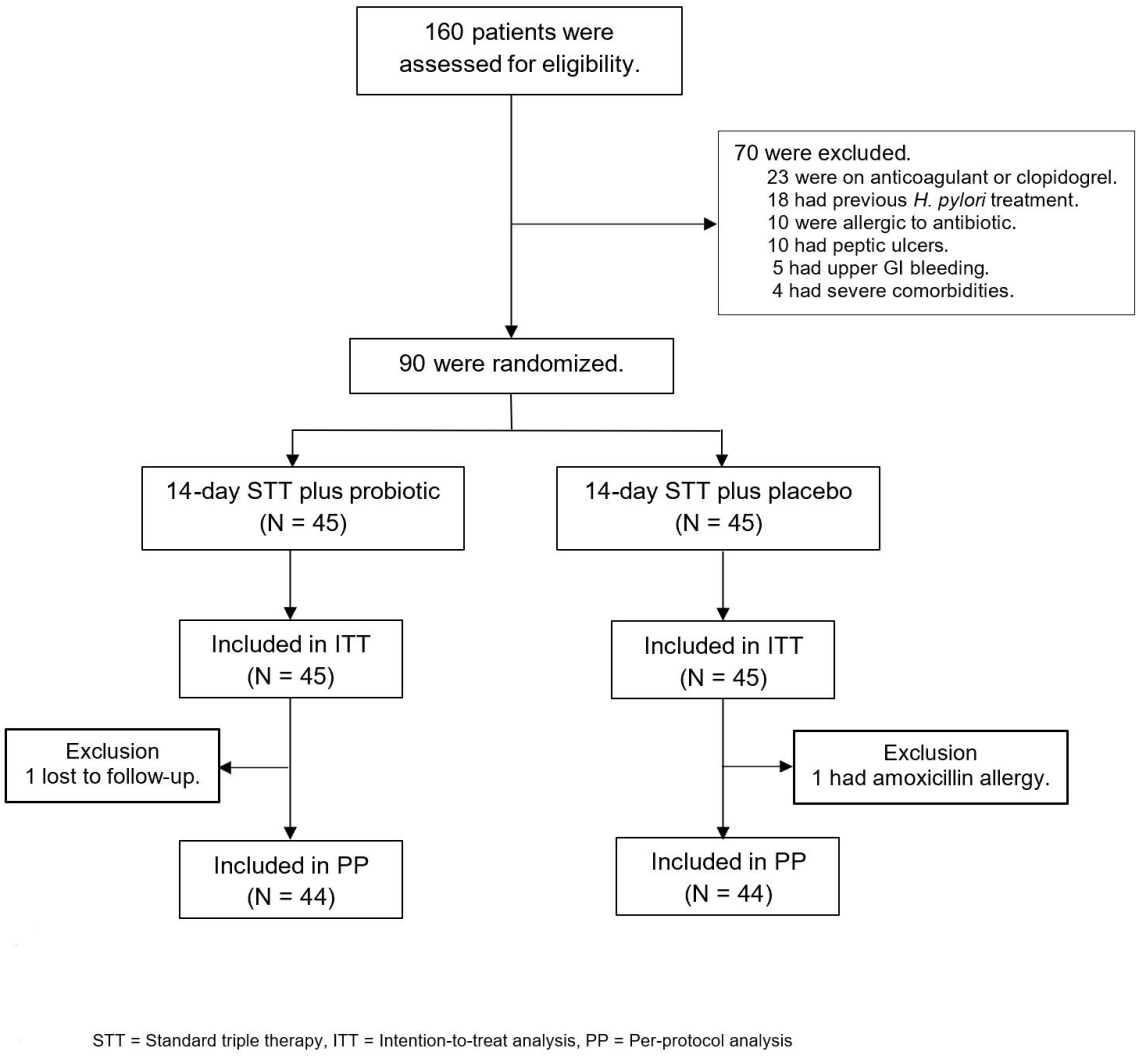
Study enrollment and treatment after randomization. STT, standard triple therapy; ITT, intention-to-treat analysis; PP, per-protocol analysis.

Out of the 160 patients with *H. pylori* infection who were assessed for eligibility, 70 were excluded and 90 were enrolled in the study and randomly assigned to receive 14-day standard triple therapy with either probiotics or placebo (**Figure 2**). Baseline demographic characteristics were similar between the groups at baseline (**Table 1**). The most common endoscopic finding was chronic non-atrophic gastritis (76.7%), followed by hemorrhagic gastritis (12.2.%) and atrophic gastritis (11.1%). Patients were diagnosed with *H. pylori* infection if they had a positive test result for one of three diagnostic methods, namely, the rapid urease test, *H. pylori* culture, and histologic examination, as demonstrated in **Table 1**. One patient in the probiotic group (lost to follow-up) and one patient in the placebo group (amoxicillin allergy) were excluded from the ITT analysis (**Figure 2**).

**Table 1:** Baseline characteristics of patients.

Characteristic	14-day STT plus probiotic treatment regimen (N = 45)	14-day STT plus placebo treatment regimen (N = 45)	*p*-value
Male, *n* (%)	20 (44.4%)	23 (51.1%)	0.527
Age (years, mean ± SD)	54.5 ± 10.5	55.3 ± 9.5	0.721
BMI (kg/m^2^, mean ± SD)	24.6 ± 5.5	23.6 ± 3.9	0.330
Underlying diseases			
Hypertension	13 (28.9%)	12 (26.7%)	0.814
Dyslipidemia	10 (22.2%)	14 (31.1%)	0.340
Diabetes mellitus	3 (6.7%)	9 (20.0%)	0.063
Smoking	11 (24.4%)	15 (33.3%)	0.352
Alcohol	14 (31.1%)	17 (37.8%)	0.506
Diagnosis of *H. pylori* infection			
Rapid urease test	29 (64.4%)	27 (60.0%)	0.664
Histopathology	44 (97.8%)	43 (95.6%)	1.000
Culture or HelicoDR	11 (24.4%)	14 (31.1%)	0.480
Endoscopic findings			
Chronic non-atrophic gastritis	35 (77.8%)	34 (75.6%)	0.803
Hemorrhagic gastritis	6 (13.3%)	5 (11.1%)	0.748
Atrophic gastritis	4 (8.9%)	6 (13.3%)	0.502

STT, standard triple therapy; BMI, body mass index.

### Eradication of *H. pylori* infection

**Figure 3:**
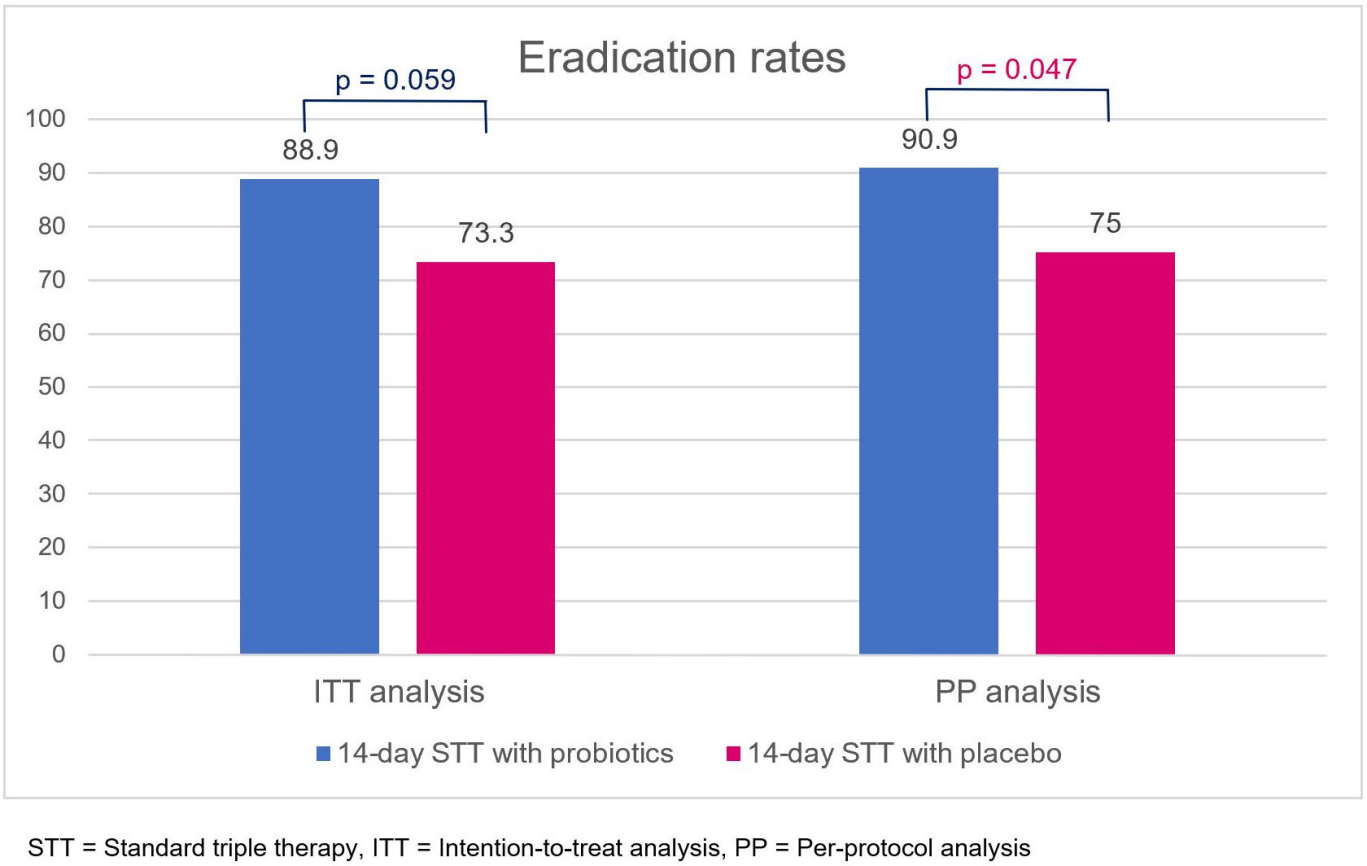
Eradication rates of 14-day standard triple therapy with either probiotics or placebo, obtained using intention-to-treat and per-protocol analyses. STT, standard triple therapy; ITT, intention-to-treat analysis; PP, per-protocol analysis.

In the ITT analysis, eradication rates of 14-day triple therapy with probiotics and placebo were 88.9% and 73.3%, respectively (*p* = 0.059). In the PP analysis, 14-day triple therapy with probiotics provided a significantly higher eradication rate than placebo (90.9% vs. 75.0%, *p* = 0.047) (**Figure 3**). Antibiotic susceptibility testing was conducted using an E-test and GenoType^®^ HelicoDR, which demonstrated a high clarithromycin resistance rate (6 out of 25, 24%). Metronidazole resistance was also high (7 out of 12, 58.3%), whereas there was no tetracycline (0 out of 12, 0%), amoxicillin (0 out of 12, 0%), and levofloxacin resistance (0 out of 25, 0% from both E-test and HelicoDR). There was no significant difference in clarithromycin-resistant strains in the probiotic and placebo groups [1 out of 14 (7.1%) vs. 5 out of 11 (45.5%), *p* = 0.056]. The subgroup analysis of clarithromycin-sensitive strains demonstrated no significant difference in eradication rates between the probiotic and placebo groups [11 out of 13 (84.6%) vs. 6 out of 6 (100%), *p* = 1.00]. For clarithromycin-resistant strains, there was no difference in the eradication rates between the probiotic and placebo group [1 out of 1 (100%) vs. 2 out of 5 (40%), *p* = 1.00].

### Gastrointestinal symptoms

**Figure 4:**
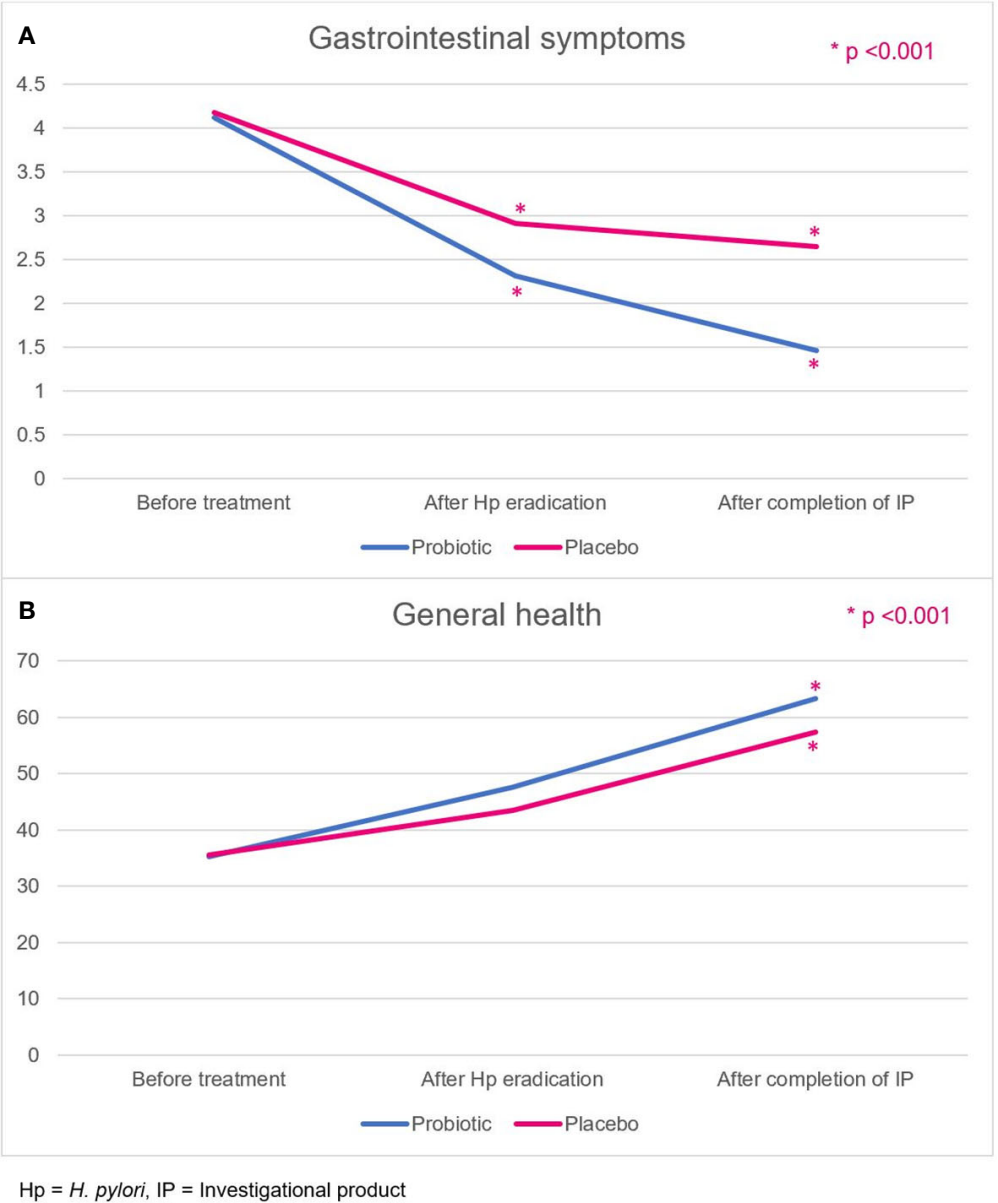
**(A)** Gastrointestinal symptoms, and **(B)** general health-related QoL scores in the probiotic and placebo groups. Hp, *H. pylori*; IP, investigational product.

The GSRS comprises five dimensions, namely, reflux, abdominal pain, indigestion, diarrhea, and constipation.^14^ Patients in the probiotic and placebo groups had comparable GSRS scores at baseline (4.12 ± 0.46 vs. 4.18 ± 0.68, *p*=0.664). However, the GSRS scores of the probiotic group were significantly improved compared with those of the placebo group (**Figure 4A**), both at week 2, after the *H. pylori* eradication therapy regimen (2.31 ± 0.45 vs. 2.91 ± 0.70, *p*<0.001), and at week 6, after completion of the probiotics or placebo course (1.46 ± 0.36 vs. 2.65 ± 0.66, *p*<0.001).

### Health-related quality of life

General health is one of the quality-of-life dimensions evaluated by the SF-36 score. Patients rated their overall current general health status, health comparison with others, and health expectation in the future. Patients in the probiotic and placebo groups had comparable general health scores at baseline (35.2 ± 14.0 vs. 35.5 ± 14.7, *p*=0.941). The scores in both groups were not different after *H. pylori* eradication (week 2; 47.6 ± 10.1 vs. 43.4 ± 10.7, *p*=0.061). After completion of probiotics or placebo (week 6), the probiotic group demonstrated a more improved general health-related quality of life score (63.3 ± 10.2 vs. 57.3 ± 13.4, *p*=0.020) than the placebo group (**Figure 4B**).

### Adverse events

Typical side effects associated with standard eradication therapy, including bloating, diarrhea, bitter taste, and nausea, were reported in both groups (**Table 2**). However, patients in the probiotic group had a significantly lower incidence of bloating, diarrhea, bitter taste, and nausea than those in the placebo group (15.9% vs. 40.9%, *p* = 0.012; 13.6% vs. 40.9%, *p* = 0.006; 4.5% vs. 25.0%, *p* = 0.015; 2.3% vs. 34.1%, *p* = 0.003, respectively). No unexpected adverse events or serious adverse events were reported in either group.

**Table 2:** Adverse events comparing between regimens with probiotic or placebo.

Adverse events	Probiotic group (N = 44)	Placebo group (N = 44)	Odds ratio (95% CI)	*p*-value
Bloating	7 (15.9%)	18 (40.9%)	0.27 (0.10 to 0.75)	0.012
Diarrhea	6 (13.6%)	18 (40.9%)	0.23 (0.28 to 0.65)	0.006
Bitter taste	2 (4.5%)	11 (25.0%)	0.14 (0.03 to 0.69)	0.015
Nausea	1 (2.3%)	15 (34.1%)	0.05 (0.01 to 0.36)	0.003

## Discussion

*H. pylori* eradication is recommended for reducing dyspeptic symptoms and preventing gastric cancer (1, 17). Standard triple therapy has been used for *H. pylori* eradication since the 1990s (18). However, over the last decade, its efficacy has dropped to 80% or less, and it is no longer recommended as a first-line treatment in regions with high clarithromycin resistance (1, 19). This randomized, double-blind, placebo-controlled study demonstrated that triple therapy with a probiotic as an adjuvant could yield an excellent eradication rate. Furthermore, reduced therapy side effects, such as gastrointestinal symptoms (i.e., bloating, diarrhea, bitter taste, and nausea) and improved quality-of-life scores were also reported in the probiotic group.

Adding probiotics to standard triple therapy significantly improved the eradication rate in the probiotic group (shown by per-protocol analysis) compared with the placebo group (90.9% vs. 75.0%, *p* = 0.047). The results of this study agree with those of previous trials in which triple therapy was used as a regimen for *H. pylori* eradication (20, 21); other studies, using different or non-viable strains of probiotics, demonstrated conflicting results (22, 23). We reviewed nine previous studies that demonstrated better eradication rates of triple therapy with probiotics than with placebo, as shown in **Supplementary Table 1** (15, 21, 24–30). Most studies used a 7-day triple therapy regimen, except for one in Croatia, which used a 14-day regimen. Each study used a different probiotic protocol (i.e., before, during, or after triple therapy). Only three studies reported high clarithromycin resistance, which was not different between the probiotic and placebo groups (21, 29, 30). The subgroup analysis of clarithromycin-resistant strains in our study revealed that there was no difference in the eradication rates between the probiotic and placebo groups, which was similar to the findings of a study conducted in Japan (eradication rates: 38.5% vs. 28.0%, respectively, *p*=0.428) (21). This emphasizes that the primary cause of treatment failure of triple therapy is clarithromycin resistance.

Antibiotic resistance is a key factor associated with *H. pylori* treatment failure (31). The high clarithromycin resistance rate (24%) in this study could have contributed to eradication failure after triple therapy. However, the eradication rate of triple therapy with probiotics in our study was still as high as 90.9% in the PP analysis. The combined effects of lowered amounts of *H. pylori* and decreased side effects because of probiotics might improve the eradication rate even for antibiotic-resistant strains. For clarithromycin-sensitive strains, added benefits of reducing adverse events were reported in the probiotic group, while excellent eradication rates (96%–100%) were already demonstrated in both probiotic and placebo groups (32). Since there was only a small number of clarithromycin-resistant strain in the probiotic arm in this study, whether or not the addition of probiotics could yield a better eradication rate for antibiotic-resistant strains than the addition of a placebo is still to be determined in future research.

Our study used probiotics during and after *H. pylori* treatment, as we presumed that probiotics could relieve adverse events while patients were undergoing antibiotic therapy and improve gut dysbiosis after *H. pylori* eradication. The two prior randomized trials from our center using single-strain probiotics reported significantly decreased adverse events in the probiotic arm, but no statistical difference in eradication rates between the probiotic and the placebo group (33, 34). However, this study showed a significantly higher eradication rate in the probiotic arm, which is in agreement with the generally superior *H. pylori* eradication effects reported for *Lactobacillus* strains or multi-strain probiotics (9, 35). A recent study reported that *L. acidophilus* and *L. rhamnosus* could directly decrease the *H. pylori* bacterial load without observed alterations in gut microbiota diversity and composition (36). In addition to conferring direct protective effects by maintaining gut barrier function, *L. rhamnosus and L. helveticus* can also reduce the incidence of antibiotic-associated diarrhea, which might explain the substantial reduction in antibiotic-related side effects, such as bloating and diarrhea, observed in the probiotic group in our study (10). After successful eradication, there will be an improvement of *H. pylori*-induced gastric microbial dysbiosis and restoration of normal microbiota (37). A previous study revealed that 14-day triple therapy could induce minimal perturbation of gut microbiota until the recovery of dysbiosis 8 weeks after eradication therapy (38). One study demonstrated that long-term dietary supplementation of *L. rhamnosus* (30 weeks) could reduce the levels of serum cytokines (tumor necrosis factor α and interleukin-10), modulate the immune response, and alter the gut microbiome. The abundance of *Helicobacteraceae* also decreased in the group treated with *L. rhamnosus.* Microbial dysbiosis might be alleviated by probiotic supplementation (39). Therefore, the probiotic treatment was extended for another 4 weeks after the completion of triple therapy as we thought that it might help to accelerate the restoration of gut microbiota and consequently relieve gastrointestinal symptoms such as bloating, nausea, and diarrhea.

The significant improvement of gastrointestinal symptoms and general health scores were also reported in the probiotic group after treatment. The improvement in the GSRS score was comparable to that reported in a previous study using triple therapy with *Lactobacillus reuteri* (40), but another trial reported no difference in the GSRS score after treatment (41). The difference among studies was that in ours, treatment with probiotics continued for a longer period (4 weeks) after the completion of *H. pylori* treatment than in other studies (2 weeks). Moreover, the trial reporting no difference in the GSRS score was a single-blind study, and no antibiotic susceptibility testing was conducted in either study. Better general health scores as one indicator of quality of life were also demonstrated in this study. Quality of life is uncommonly evaluated in the study of *H. pylori* treatment with probiotics. There was only one study reporting improved quality of life by SF-36 after a 2-week *H. pylori* eradication regimen (42). Our study exhibited better improvement of general health in the probiotic group after completion of *H. pylori* treatment and probiotics (week 6).

In conclusion, 14-day standard triple therapy with probiotic supplement yielded an excellent *H. pylori* eradication rate compared with that observed in the placebo group, which was suboptimal. Adding probiotics as an adjuvant to standard triple therapy also reduced antibiotic-associated adverse events, and improved patients’ gastrointestinal symptom scores and their general health-related quality of life after treatment.

## Data availability statement

The raw data supporting the conclusions of this article will be made available by the authors, without undue reservation.

## Ethics statement

The studies involving humans were approved by the Human Research Ethics Committee of Thammasat University, Thailand. The studies were conducted in accordance with the local legislation and institutional requirements. The participants provided their written informed consent to participate in this study.

## Author contributions

Conceptualization: AK and RV. Data curation: AK, NA, and RV. Formal analysis: AK. Funding acquisition: RV. Investigation: AK and NA. Methodology: AK, NA, and RV. Project administration: RV. Resources: NA and RV. Software: AK. Supervision: VM and RV. Validation: AK, NA, and RV. Visualization: AK and NA. Writing—original draft: AK and NA. Writing—review and editing: BP, SC, SS, PB, PN, NI, VM, and RV. All authors contributed to the article and approved the submitted version.
