# Supplementary material for: Efficacy and safety of Lacticaseibacillus rhamnosus R0011 and Lactobacillus helveticus R0052 as an adjuvant for Helicobacter pylori eradication: a double-blind, randomized, placebo-controlled study
Source: Front Gastroenterol (Lausanne). 2023 Sep 14;2:1245993. doi: 10.3389/fgstr.2023.1245993 (PMC12952351; doi:10.3389/fgstr.2023.1245993)
Supplement: Supplementary file 1 [file Table_1.docx]

**Supplementary table 1.** Comparison of studies with improved eradication rates using triple therapy with probiotics

| **Study, year** | **Location** | **Total patients**  **(probiotic/control)** | **Duration of triple therapy (days)** | **Probiotic strains** | **Time of probiotics** | **Duration of probiotic (weeks)** | **Eradication rates**  **(probiotic vs. control, p-value)** |
| --- | --- | --- | --- | --- | --- | --- | --- |
| Sheu, 2002 | Taiwan | 160 (80/80) | 7 | *Lactobacillus Bifidobacterium* | Same and after | 5 | 91.3% vs. 78%, p=0.045 |
| De Bortoli, 2007 | Italy | 206 (105/101) | 7 | *Lactobacillus spp.*  *Bifidobacterium*  *S. thermophilus* | Same | 1 | 88.6% vs. 72.3%, p=0.005 |
| Song, 2010 | Korea | 991 (330/331) | 7 | *S. boulardii* | Same and after | 4 | 80% vs. 71.6%, p=0.003 |
| Deguchi, 2012 | Japan | 229 (115/114) | 7 | *L. gasseri* | Before and same | 4 | 82.6% vs. 69.3%, p=0.018 |
| Du, 2012 | China | 234 (155/79) | 7 | *L. acidophilus*  *S. faecalis*  *B. subtilis* | Gr 1: before  Gr 2: after  Gr 3: no probiotic | 2  2  0 | (Gr 1) 79.5% vs. (Gr 2) 79.2% vs. (Gr 3) 60.8%, p=0.014 (Gr1vs3), p=0.015 (Gr2vs3) |
| Chitapanarux, 2015 | Thailand | 63 (31/32) | 7 | *B. longum* | Same and after | 4 | 90.32% vs. 68.79%, p=0.034 |
| Hauser, 2015 | Croatia | 650 (333/317) | 14 | *L. rhamnosus GG Bifidobacterium* | Same | 2 | 87.38% vs. 72.55%, p<0.001 |
| Tongtawee, 2015 | Thailand | 300 (200/100) | 7 | *L. delbrueckii*  *S. thermophilus* | Gr 1: before  Gr 2: before and after  Gr 3: no probiotic | 1  2  0 | (Gr 1) 77.3% vs. (Gr 2) 80.4% vs. (Gr 3) 74.5%, p<0.01 |
| Tongtawee, 2015 | Thailand | 200 (100/100) | 7 | *L. delbrueckii*  *S. thermophilus* | Before | 1 | 89% vs. 81%, p=0.01 |

Gr = Group, wk = week, Time of probiotics: before = before triple therapy, same = same period as triple therapy, after = after triple therapy
